# Recurrent fusion transcripts in squamous cell carcinomas of the vulva

**DOI:** 10.18632/oncotarget.15167

**Published:** 2017-02-07

**Authors:** Marta Brunetti, Antonio Agostini, Ben Davidson, Claes G Tropé, Sverre Heim, Ioannis Panagopoulos, Francesca Micci

**Affiliations:** ^1^ Section for Cancer Cytogenetics, Institute for Cancer Genetics and Informatics, The Norwegian Radium Hospital, Oslo University Hospital, Oslo, Norway; ^2^ Centre for Cancer Biomedicine, University of Oslo, Oslo, Norway; ^3^ Department of Pathology, The Norwegian Radium Hospital, Oslo University Hospital, Oslo, Norway; ^4^ Faculty of Medicine, University of Oslo, Oslo, Norway; ^5^ Department of Gynecology, The Norwegian Radium Hospital, Oslo University Hospital, Oslo, Norway

**Keywords:** fusion gene, promoter swapping, squamous cell carcinoma, transcriptome sequencing, PCR

## Abstract

Juxtaposition of two different genes or gene parts due to chromosomal rearrangement is a well-known neoplasia-associated pathogenetic mechanism. The detection and characterization of such tumorigenic fusions is of great importance both research-wise, diagnostically because they may be specific for distinct tumor entities, and because they may serve as therapeutic targets for antioncogenic drugs that interact directly with the molecular changes responsible for neoplastic transformation.

At present, more than 10,000 fusion transcripts have been reported in different types of neoplasia, with one tenth of them being identified in squamous cell carcinomas (SCC) of different locations. No recurrent fusion gene has to date been identified in SCC of the vulva.

We performed high-throughput paired-end RNA-sequencing of 12 vulvar SCC and found two recurrent fusions with the *STIP1*-*CREB3L1* and *ZDHHC5*-*GPR137* being present in two tumors each. The transcripts were detected only in the tumor samples, not in normal vulvar tissue from healthy donors used as control. The *CREB3L1* and *ZDHHC5* genes encode proteins involved in transcription suggesting that the chimeras may alter downstream events in their respective pathways. Expression analysis of the *CREB3L1* gene showed the presence of two distinct groups of tumors, one having fusion and downregulation of the gene and the other showing upregulation of *CREB3L1*.

## INTRODUCTION

Vulvar cancer is the fourth most common gynecologic cancer accounting for 5% of all malignancies of the female genital tract. Squamous cell carcinoma (SCC) is the most common vulvar malignancy (95%) followed by melanoma, sarcoma, and basal cell carcinoma [1].

About 40 (*n* = 44) vulvar SCC have been karyotyped and scientifically reported until now [2–5]. Chromosome-based comparative genomic hybridization (CGH) has been performed on less than 40 tumors [4, 6, 7] while array-based CGH data are limited to 13 samples [5]. The most common changes have involved chromosomes 3 and 8. On chromosome 3, a deletion of the fragile histidine triad (*FHIT*) gene in 3p14 was reported leading to downregulation of gene expression [5]. Lately, overexpression of the high-mobility AT-hook 2 (*HMGA2*) gene, mapping on 12q14.3, was shown [8].

Since the cytogenetic and molecular data on SCC of the vulva are so few and no specific aberrations have been identified as the main event in tumorigenesis or tumor progression, we decided to apply the latest methodology of transcriptome sequencing, high-throughput paired-end RNA-sequencing, to investigate whether fusion genes are present in SCC of the vulva.

The formation of a fusion gene is often a crucial pathogenetic event in neoplastic transformation. Different neoplasias typically have different fusion genes. The identification of such fusion transcripts therefore not only holds the key to an understanding of tumorigenesis, but may also be diagnostically and prognostically important and the qualitatively new gene or its product may even be the target for new antioncogenic therapeutics. Cancer-specific fusion genes have been found in hematological malignancies, mesenchymal tumors, and carcinomas in increasing numbers. Around 1,500 fusion transcripts have been reported in SCC of different sites (Mitelman Database of Chromosome Aberrations and Gene Fusions in Cancer at http://cgap.nci.nih.gov/Chromosomes/Mitelman; update February 2016); however, no studies have addressed the possible presence of such genes in SCC of the vulva.

## RESULTS

**Figure 1:**
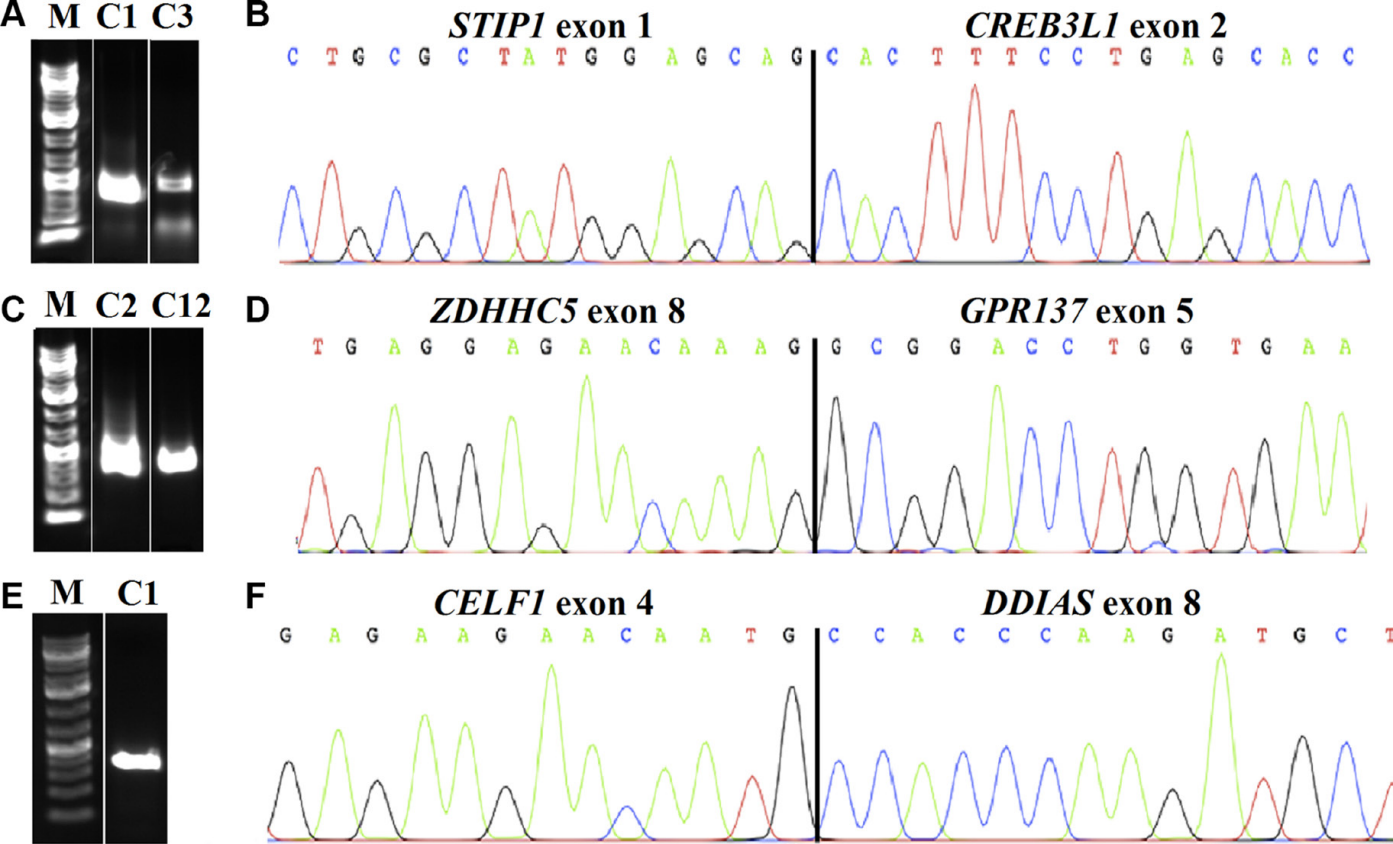
Detection of fusion transcripts in SCC of the vulva The chimeric transcripts were amplified with primer combinations STIP1F1/CREB3L1R2 in cases 1 (C1) and 3 (C3) (**A**), ZDHHC5F2/GPR137R2 in cases 2 (C2) and 12 (C12) (**C**), and CELF1F2/C11orf82R2 in case 1 (C1) (**E**), M: 1Kb DNA ladder. Partial chromatogram showing the junction of the *STIP1-CREB3L1* (**B**); *ZDHHC5-GPR137* (**D**); and *CELF1-C11orf82* (**F**).

All 12 vulvar SCC sent for transcriptome sequencing gave informative results. An average of 101 (range 16-503) possible fusion transcripts was obtained per tumor. Since some of the putative fusions offered by this algorithm probably are false positives [9], we manually executed a BLAT command to identify those with 100% identity in the genome. We ended up with 10 putative fusions from eleven cases; case 9 showed no fusion. Their real presence was tested for by PCR followed by Sanger sequencing (direct sequencing). In all cases, a specific PCR product was found. An overview of the fusions, tumors, and breakpoint positions is given in Table 1. The possible presence of these fusion transcripts was also tested for in the four normal vulva tissue controls. Three of the fusions were not found in these controls, namely *STIP1-CREB3L1*, *ZDHHC5-GPR137*, and *CELF1-DDIAS*. These transcripts had been found in case 1 (*STIP1-CREB3L1* and *CELF1-DDIAS*) whereas *ZDHHC5-GPR137* was found in case 2; they were then tested for in all SCC available (12 samples in total). *STIP1-CREB3L1* and *ZDHHC5-GPR137* were found in one additional tumor each, in cases 3 and 12, respectively. The three here identified transcripts fuse exon 1 of the stress-induced phosphoprotein 1 (*STIP1*; accession number NM_006819.2) with exon 2 of the cAMP responsive element binding protein 3-like 1 (*CREB3L1*; accession number NM_052854.3), exon 8 of the zinc finger *ZDHHC5* (accession number NM_015457.2) with exon 5 of the G protein receptor *137* (*GPR137*; accession number NM_001170881.1), and exon 4 of the CUGBP Elav-like family member 1 (*CELF1*; accession number NM_006560.3) with exon 8 of the DNA damage-induced apoptosis suppressor (*DDIAS*; previously known as *C11orf82*; accession number NM_145018; sequence AKO58145.1, which has been found so far only in testis) (Figure 1). In each transcript, the fusion is in-frame.

**Table 1:** Overview of the SCC included in the study, their diagnosis, the fusion transcripts tested for each of them, the HPV status, and the immunohistochemistry data

Case/lab number	Diagnosis	Fusion transcripts tested	Chromosomal location gene 1-gene 2	HPV genotyping	Immunohistochemistry score
1/02-91	SCC moderately differentiated	*PPFIA1-AHNAKIRF6-C1orf74* *IRF6-C1orf74 STIP1-CREB3L1* *CELF1-DDIAS*	11q13.3-11q12.3 1q32.2-1q32.2 11q13.1-11p11.2 11p11.2-11q14.1	−	3
2/02-167	SCC moderately differentiated	*ZDHHC5-GPR137* *IRF6-C1orf74* *RBM4-SIPA1*	11q12.1-11q13.1 1q32.2-1q32.2 11q13.2-11q13.1	−	2
3/02-848	SCC moderately differentiated	*WNK1-B4GALNT3* *IRF6-C1orf74*	12p13.33-12p13.33 1q32.2-1q32.2	−	0
4/02-869	SCC*	*IRF6-C1orf74* *ADCK4-NUMBL*	1q32.2-1q32.2 19q13.2-19q13.2	−	1
5/03-830	SCC moderately differentiated	*ADCK4-NUMBL* *IRF6-C1orf74* *MFSD3-LRRC14*	19q13.2-19q13.2 1q32.2-1q32.2 8q24.3-8q24.3	−	1
6/03-1011	SCC*	*IRF6-C1orf74* *ADCK4-NUMBL*	1q32.2-1q32.2 19q13.2-19q13.2	−	2
7/03-1088	SCC well differentiated	*IRF6-C1orf74*	1q32.2-1q32.2	n.a.**	—
8/006-19	SCC poorly differentiated	*IRF6-C1orf74*	1q32.2-1q32.2	−	2
9/06-125	SCC moderately differentiated	--		−	1
10/06-709	SCC moderately differentiated	*IRF6-C1orf74*	1q32.2-1q32.2	HPV 16	3
11/09-0733	SCC well differentiated	*DDX5-POLG2IRF6-C1orf74*	17q23.3-17q23.3 1q32.2-1q32.2	−	1
12/09-818	SCC moderately differentiated	*PLCB3-BAD* *IRF6-C1orf74* *PARP12-SLC37A3*	11q13.1-11q13.1 1q32.2-1q32.2 7q34-7q34	−	0

*information on grade is not available.

**n.a.= not available.

**Figure 2:**
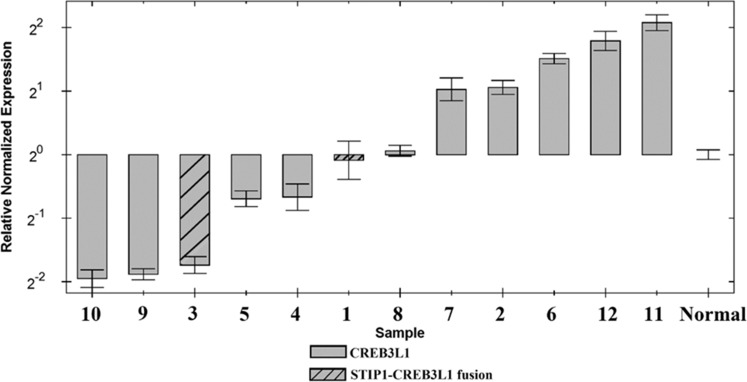
Expression profiles for the *CREB3L1* gene in 12 SCC of the vulva and in normalized control samples

Quantitation of the expression of *CREB3L1* in all SCC showed the tumors distributed into two groups: cases 1, 3, 4, 5, 9, and 10 had the gene downregulated whereas cases 2, 6, 7, 8, 11, and 12 had it upregulated (Figure 2). Both tumors with *STIP1-CREB3L1* fusion (cases 1 and 3) showed downregulation of the gene.

**Figure 3:**
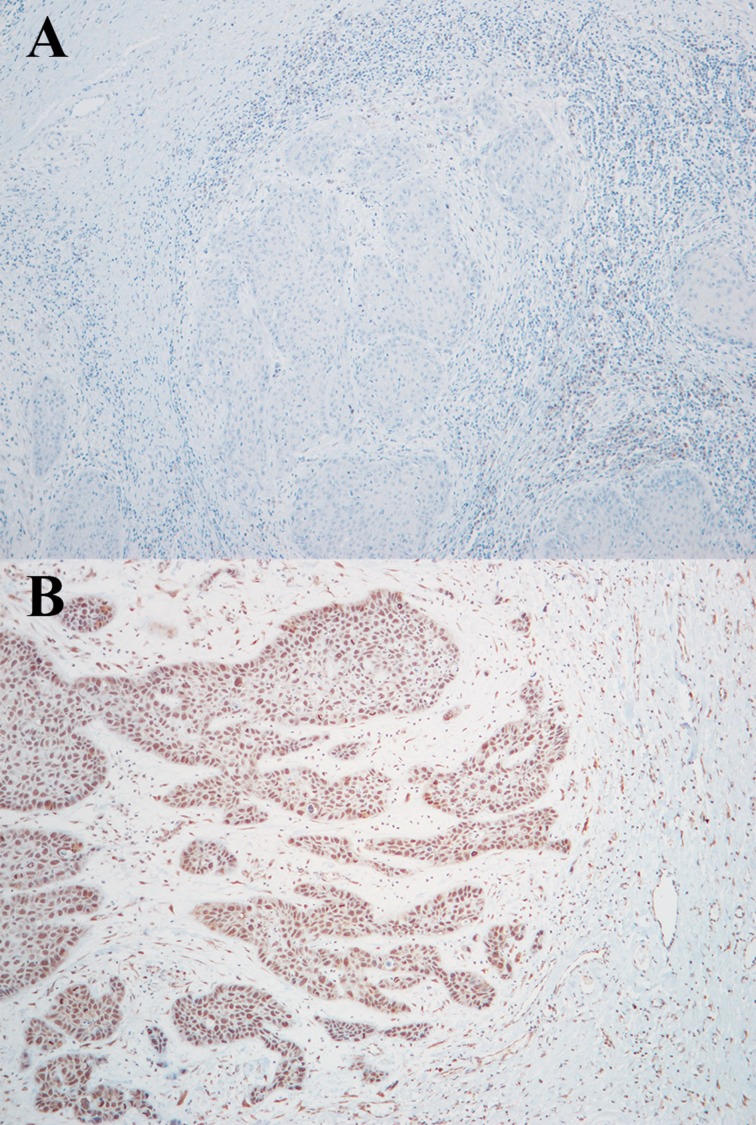
Immunohistochemistry assay for CREB3L1 protein expression in case 3 (**A**: score 0) and case 6 (**B**: score 2).

Immunohistochemistry for expression of CREB3L1 showed a score value from 0 to 3 for each tumor (Table 1; Figure 3). None of the 11 tumors available for HPV genotyping show HPV infection except for case 10 which had HVP 16 (Table 1).

## DISCUSSION

Fusion genes constitute a class of mutations in cancer that has attracted a lot of attention lately as they not only tell us something about the underlying pathogenetic processes, but also because they may provide targets for therapeutic drugs [10]. The number of chimeric transcripts thus detected has increased exponentially after the introduction of next generation sequencing (NGS) methodology. To date, the Mitelman Database of Chromosome Aberrations and Gene Fusions in Cancer (http://cgap.nci.nih.gov/Chromosomes/Mitelman) reports 10.277 fusion transcripts (update February 2016) found in different types of tumors, of which 1.430 were identified in SCC of different locations. To our knowledge, this is the first report on recurrent chimeric transcripts in vulvar SCC. The NGS analysis of 12 tumors identified three fusion transcripts, *STIP1-CREB3L1*, *ZDHHC5-GPR137*, and *CELF1-DDIAS*, of which the first two were recurrent as they were found in two different tumors, whereas the third was identified in only one. None of the three was identified in four samples of normal vulva tissue from healthy donors used as controls, underlining their specificity.

In the *STIP1-CREB3L1* chimeric transcript, the 5′ gene (*STIP1*) encodes an adaptor protein that coordinates the functions of heat shock proteins (HSP) 70 and HSP90 in protein folding. It is thought to assist in the transfer of proteins from HSP70 to HSP90 by binding both HSP90 and substrate-bound HSP70. STIP1 also stimulates the ATPase activity of HSP70 and inhibits the ATPase activity of HSP90, suggesting that it regulates both the conformations and ATPase cycles of these chaperones (https://genome.ucsc.edu/). The 3′ gene of the fusion, *CREB3L1*, encodes a protein normally found in the membrane of the endoplasmic reticulum (ER). Upon stress to the ER, the encoded protein is cleaved and the released cytoplasmic transcription factor domain translocates to the nucleus. There it activates the transcription of target genes by binding to box-B elements (https://genome.ucsc.edu/). The chimeric fusion retains only three amino acids from exon 1 of the *STIP1* gene indicating that the genomic rearrangement brings *CREB3L1* under the influence of *STIP1* regulatory elements, with promoter swapping leading to deregulation of the 3′ gene [11] [12]. Due to the fusion, the CREB3L1 protein misses half (34 out of 60 aa; NP_443086.1) of its Transcription Activation region; however, it retains the basic Leucine Zipper Domain (bZIP). Real-Time PCR showed that both tumors with the *STIP1-CREB3L1* fusion had lower expression of the *CREB3L1* gene compared to the normal controls. Also four additional SCC were similar to the tumors containing the fusion in that they were downregulated (Figure 2). We believe that this is an example of promoter swapping leading to the expression of *CREB3L1* being regulated by another promoter belonging to *STIP1* in cases 1 and 3 but via an unknown mechanism in the other four SCC. Unfortunately, no genome sequencing could be performed on these tumors. Deregulation of gene expression through gene fusion and/or promoter swapping is a well known mechanism behind tumorigenesis and tumor progression [11, 12], but this is the first time that evidence of *CREB3L1* downregulation is found in vulvar SCC. The *STIP1* gene was previously found rearranged with *AHNAK* in adenocarcinoma of the lung [13]; however, in that particular case *STIP1* constituted the 3′ moiety of the fusion whereas in the present case it is 5′.

*CREB3L1* is a gene well known for its involvement in chimeric transcripts in soft tissue tumors. It has been reported to be fused with *EWSR1* in sclerosing epithelioid fibrosarcomas [14, 15] and low-grade fibromyxoid sarcomas [16, 17], and with *FUS* in low-grade fibromyxoid sarcomas [18, 19]. Furthermore, an *EWSR1-CREB3L1* fusion was found in small cell osteosarcoma of the skeleton [20]. In all these instances, *CREB3L1* was the 3′ part of the fusion as it is in our two SCC of the vulva; however, with different breakpoints.

The second recurrent and specific fusion here identified was *ZDHHC5-GPR137. ZDHHC5* (the 5′ gene) codes for a zinc finger with a protein domain that acts as an enzyme (DHHC domain) required for palmitoyl transferase activity whereas the *GPR137* (3′ gene) codes for a G protein receptor (https://genome.ucsc.edu/). The chimeric protein retains the zf-DHH domain from the 5′ gene and 154 aa downstream from the 3′ gene. *ZDHHC5* was previously seen involved in neoplasia with different partners: a *ZDHHC5-SMTNL1* fusion was found in SCC of the ovary, a *CTNND1-ZDHHC5* in adenocarcinoma of the lung, *ZDHHC5-LCT* in adenocarcinoma of the breast, and *MEF2D-ZDHHC5* in grade III-IV astrocytoma of the brain [13]. The *GPR137* gene has also been identified in two fusions: with *PAFAH1B2* and *USP32* in adenocarcinoma of the lung and of the breast, respectively [13].

The last fusion, found in only one SCC of the vulva (case 1), was between *CELF1* and the DNA damage-induced apoptosis suppressor *DDIAS* gene. *CELF1* encodes a member of the CELF/BRUNOL family of RNA-binding proteins implicated in the regulation of several post-transcriptional events such as pre-mRNA alternative splicing, mRNA editing, mRNA translation, and stability. They mediate exon inclusion and/or exclusion in pre-mRNAs that are subject to tissue-specific and developmentally regulated alternative splicing. The fusion protein contains two N-terminal RNA recognition motif (RRM) domains, one C-terminal RRM domain, and a divergent segment of 160-230 aa between the second and third RRM domains. The chimeric protein retains the RRM1 and part of the RRM2 conserved domains from CELF1 but no domain from DDIAS. *CELF1* has been found involved in previous fusions with five different partners: with *MTCH2* and *MYOM1* in SCC and adenocarcinoma of the lung, respectively; with *LUZP2* and *ARFGAP2* in adenocarcinoma of the breast; and with *BBOX1* in grade I-II astrocytoma of the brain [13]. According to the Mitelman Database of Chromosome Aberrations and Gene Fusions in Cancer (http://cgap.nci.nih.gov/Chromosomes/Mitelman), the *DDIAS* gene was not found involved in any fusion prior to the present report.

Interestingly, both *STIP1-CREB3L1* and *CELF1-DDIAS* were identified in the same tumor (case 1). We hypothesize that the former is the primary event in tumorigenesis of SCC of the vulva since it was present also in another tumor, whereas the latter is more likely to represent a secondary event.

All three fusions reported here for the first time involve genes that involve transcription: the proteins encoded by *CREB3L1* and *ZDHHC5* contain leucine zipper domains or have zinc fingers, both of which define common classes of transcription factors, whereas CELF1 belongs to a family of proteins implicated in the regulation of several post-transcriptional events. It is known that in a subset of fusion genes often generated by recurrent chromosome aberrations, one or both genes code for transcription factors/regulators. The resulting fusion proteins have aberrant transcriptional function compared to their wild-type counterparts. These fusion transcription factors disrupt multiple biological pathways by altering expression of target genes resulting in changed cellular behavior that contributes to the tumorigenic process. The importance of such chimeric transcripts is well documented especially in sarcomas [10, 21]. The present findings indicate that alteration of one or more gene(s) important for transcription plays a role also in the tumorigenesis and/or progression of SCC of the vulva. The fact that the fusions *STIP1-CREB3L1*, *ZDHHC5-GPR137*, and *CELF1-DDIAS* were present in only four out of 12 tumors suggests that also additional fusions may characterize this tumor type. We performed a search of the data obtained by the FusionCatcher program using the “grep” command [9] to look for other fusion partners for *CREB3L1*, *ZDHHC5*, and *CELF1*; however, none was found. Furthermore, the fact that ten out of 11 tumors were HPV free, underlines the primary role of chromosomal abnormalities in this tumor type.

Further studies are needed to identify additional fusions as well as the frequency of those presently described. The final goal will be to identify a therapeutic drug specific for the chimeric fusions. Even in the absence of such specific treatments, however, the fusion transcripts could be used as a biomarker in patients with SCC of the vulva. All this is of necessity still far into the future, admittedly, but exemplifies in which direction future research on this tumor type may proceed.

## MATERIALS AND METHODS

### Tumor material

The material consisted of fresh samples from 12 SCC arising in the vulva and surgically removed at The Norwegian Radium Hospital between 2002 and 2009 (Table 1). The tumors have previously been characterized for their genomic alterations and deregulation of gene expression [4, 5, 8]. Four samples of normal vulva tissue from healthy donors were used as controls. The tumor biobank has been registered according to national legislation and the study has been approved by the Regional Committee for Medical Research Ethics South-East, REK; project numbers S-07194a and 2.2007.425.

### RNA isolation and cDNA synthesis

Total RNA was extracted using miRNeasy kit (Qiagen, Hilden, Germany) and QIAcube (Qiagen) from frozen tissue of 12 vulvar SCC stored at – 80°C. The quantity and quality of RNA were measured using a spectrophotometer QIAxpert System (Qiagen). For cDNA synthesis, 1 μg of total RNA was reverse-transcribed in a 20 μL reaction volume using iscript Advanced cDNA synthesis Kit for RT-PCR according to the manufacturer's instructions (Bio-Rad Laboratories, Oslo, Norway).

### RNA sequencing

RNA of good quality and concentration was available from all SCC of the vulva. A total of 3 μg of RNA per tumor was sent for high-throughput paired-end RNA-sequencing to the Norwegian Sequencing Centre at Ullevål Hospital (http://www.sequencing.uio.no/). The sequencing was performed using an Illumina HiSeq 2000 instrument. The Illumina software pipeline was used to process image data into raw sequencing data. Only sequence reads marked as “passed filtering” were used in the downstream data analysis. The FASTQC software was used for quality control of the raw sequence data (http://www.bioinformatics.babraham.ac.uk/projects/fastqc/). An average number of 89 million sequence reads (range 73 to 156 millions) was obtained from transcriptome sequencing. We used the Fusion Catcher software (version 0.99.3a beta-April 15, 2014) with the associated ENSEMBL, UCSC, and RefSeq databases automatically downloaded by FusionCatcher (https://code.google.com/p/fusioncatcher/) [22] to detect fusion transcripts.

### Reverse Transcriptase-Polymerase Chain Reaction (RT-PCR) and Sanger sequencing

cDNA equivalent to 10 ng RNA was amplified using Takara Premix Ex Taq (Takara-Bio, Europe/SAS, Saint-Germain-en-Laye, France). All primers used in the PCR reactions are listed in Table 2. The primer combinations STIP1F1/CREB3L1R1, ZDHHC5F1/GPR137R1, and CELF1F1/C11orf82R1 were used for first PCR reaction. The primer sets STIP1F1/CREB3L1R2, ZDHHC5F2/GPR137R2, and CELF1F2/C11orf82R2 were used for nested-PCR. The PCRs were run on a C-1000 Thermal cycler (Biorad) with an initial denaturation at 94°C for 30 sec, followed by 35 cycles at 98°C for 7 sec and at 55°C for 30 sec, and a final extension at 72°C for 5 min. Expression of the housekeeping gene *ABL1* was monitored as an internal control. Three μL of the PCR products were stained with GelRed (Biotium, Hayward, CA, USA) and analyzed by electrophoresis through 1.0 % agarose gel. The gel was scanned with G-Box (Syngene, Los Altos, CA, USA) and the image was acquired using GeneSnap (Syngene). The remaining amplified fragment (22 μl) was purified using the QIAquick PCR Purification kit (Qiagen) and direct sequencing was performed using a 3500 Genetic Analyzer (Applied Biosystems). The BLAST (http://blast.ncbi.nlm.nih.gov/Blast.cgi) and BLAT (http://genome.ucsc.edu/cgi-bin/hgBlat) programs were used for computer analysis of sequence data.

**Table 2:** Primers used for PCR and sequencing

Oligo designation	Sequence	Position	Gene	Accession number
STIP1F1	5′-CTTCTAGTAGGTTCCAGAAGGC-3′	59–80	STIP1	NM_006819.2
CREB3L1R1	5′-ACCATGATGGAGCACAGTTT-3′	838–857	CREB3L1	NM_052854.3
CREB3L1R2	5′-CATCTTGATGGGCACAAGGG-3′	770–789	CREB3L1	NM_052854.3
ZDHHC5F1	5′-GGATTTCACGTGGTTCTGGTG-3′	1869–1889	ZDHHC5	NM_015457.2
ZDHHC5F2	5′-CGTGTTCTCTGCAGTTCTCC-3′	1980–1999	ZDHHC5	NM_015457.2
GPR137R1	5′-GCCTAGACTGCCCGACATAC-3′	1464–1483	GPR137	NM_001170881.1
GPR137R2	5′-GCTCAGGTCCTGTGGGG-3′	1332–1348	GPR137	NM_001170881.1
CELF1F1	5′-AAAGAAAATGAACGGCACCC-3′	253–272	CELF1	NM_006560.3
CELF1F2	5′-TGCTGTGTATGAAATCAACGTC-3′	379–400	CELF1	NM_006560.3
C11orf82R1	5′-GGCCATTTGACAAAGTGAGTG-3′	1129–1149	DDIAS	AK058145.1
C11orf82R2	5′-CAAACAGGGGAGATTCACCA-3′	1055–1074	DDIAS	AK058145.1

### Real-time polymerase chain reaction (Real-time PCR)

The expression of the *CREB3L1* gene was assessed with Real-Time PCR using the TaqMan Gene Expression assay CREB3L1 (Applied Biosystems, Foster City, CA, USA) Hs_00999639_m1 (CREB3L1 exons 4–5).

The assay ACTB (Hs_01060665_g1) was used as a reference. The PCR analyses were performed using the CFX96 Touch Real-Time PCR detection system (Bio-Rad Laboratories, Oslo, Norway). The reactions were carried out in quadruplicate using the TaqMan Universal Master Mix II with UNG (Applied Biosystems) following the manufacturer's protocol. Human Universal Reference Total RNA (Clontech, Mountain View, CA, USA) was used as internal reaction control with one sample of normal vulva tissue being used for normalization.

### Immunohistochemistry

Formalin-fixed, paraffin-embedded sections were analyzed for CREB3L1 protein expression using the Dako EnVision™ Flex+ System (K8012; Dako, Glostrup, Denmark). Epitope unmasking was carried out in a low pH solution (pH 6). Sections were incubated with a 0.3% hydrogen peroxide (H_2_O_2_) solution for 5 min to block endogenous tissue peroxidase activity. Sections were then incubated with a polyclonal CREB3L1 primary antibody (Novus Biologicals, Littleton, CO; cat. # NBP1-82503) and treated with EnVision™ Flex+ mouse linker (15 min) and EnVision™ Flex/HRP enzyme (30 min), stained for 10 min with 3`3 diaminobenzidine tetrahydrochloride (DAB), counterstained with hematoxylin, dehydrated, and mounted in Richard-Allan Scientific Cyto seal XYL (Thermo Fisher Scientific, Waltham, MA). Positive control consisted of normal pancreas.

Staining was scored as negative (0% stained cells), focally positive (1–10% stained cells), moderately positive (11–50% stained cells), or diffusely positive (staining in > 50% cells), corresponding to a score of 0–3.

### DNA extraction and HPV genotyping

DNA was extracted from formalin-fixed, paraffin-embedded tissue according to a previously published protocol [23]. Quantitative polymerase chain reaction (qPCR) assay was performed on the Roche Lightcycler 480 II using Roche probe master (Roche Diagnostics, Basel, Switzerland) reagents according to the manufacturer's guidelines. HPV analysis was performed for 15 high-risk HPV types (HPV 16, 18, 31, 33, 35, 39, 49, 45, 51, 52, 56, 58, 59, 66, 68a and 68b) as well as for two low-risk types (HPV 6 and 11). Human genomic DNA was co-amplified and used as internal control.
